# Desaturation-Distance Ratio During Submaximal and Maximal Exercise Tests and Its Association With Lung Function Parameters in Patients With Lymphangioleiomyomatosis

**DOI:** 10.3389/fmed.2021.659416

**Published:** 2021-07-29

**Authors:** Douglas Silva Queiroz, Cibele Cristine Berto Marques da Silva, Alexandre Franco Amaral, Martina Rodrigues Oliveira, Henrique Takachi Moriya, Carlos Roberto Ribeiro Carvalho, Bruno Guedes Baldi, Celso R. F. Carvalho

**Affiliations:** ^1^Departament of Physical Therapy, School of Medicine, University of Sáo Paulo, Sáo Paulo, Brazil; ^2^Hospital Israelita Albert Einstein, Sáo Paulo, Brazil; ^3^Divisao de Pneumologia, Instituto do Coracao, Hospital das Clínicas da Faculdade de Medicina da Universidade de Sáo Paulo, Sáo Paulo, Brazil; ^4^Biomedical Engineering Laboratory, Escola Politécnica, Universidade de Sáo Paulo, Sáo Paulo, Brazil

**Keywords:** lymphangioleiomyomatosis, exercise tests, respiratory function tests, lung volume measurements, lung diseases

## Abstract

**Background:** The desaturation–distance ratio (DDR), the ratio of the desaturation area to the distance walked, is a promising, reliable, and simple physiologic tool for functional evaluation in subjects with interstitial lung diseases. Lymphangioleiomyomatosis (LAM) is a rare neoplastic condition frequently associated with exercise impairment. However, DDR has rarely been evaluated in patients with LAM.

**Objectives:** To assess DDR during maximal and submaximal exercises and evaluate whether DDR can be predicted using lung function parameters.

**Methods:** A cross-sectional study was conducted in a cohort of women with LAM. The 6-min walking test (6MWT) and the incremental shuttle walking test (ISWT) were performed, and DDR was obtained from both tests. The functional parameters were assessed at rest using spirometry and body plethysmography. The pulmonary function variables predictive of DDR were also assessed.

**Results:** Forty patients were included in this study. The mean age was 46 ± 10 years. Airway obstruction, reduced DL_CO_, and air trapping were found in 60, 57, and 15% of patients, respectively. The distance walked and the DDR for the 6MWT and ISWT were, respectively, 517 ± 65 and 443 ± 127 m; and 6.6 (3.8–10.9) and 8.3 (6.2–12.7). FEV_1_ (airway obstruction) and reduced DL_CO_ and RV/TLC (air trapping) were independent variables predictive of DDR during exercises field tests [DDR_6MWT_ = 18.66–(0.06 × FEV_1%pred_)–(0.10 × DL_CO%pred_) + (1.54 × air trapping), $R_{\text{adjust}}^{2}$ = 0.43] and maximal [DDR_ISWT_ = 18.84–(0.09 × FEV_1%pred_)–(0.05 × DL_CO%pred_) + (3.10 × air trapping), $R_{\text{adjust}}^{2}$ = 0.33].

**Conclusion:** Our results demonstrated that DDR is a useful tool for functional evaluation during maximal and submaximal exercises in patients with LAM, and it can be predicted using airway obstruction, reduced DL_CO_, and air trapping.

## Introduction

Lymphangioleiomyomatosis (LAM) is a rare neoplastic cystic lung disease that affects, mainly women, ~5 persons per million (1). It is characterized by the proliferation of abnormal smooth muscle-like LAM cells, resulting in vascular and airway obstruction and cyst formation (2). LAM's main clinical features are progressive dyspnea, pneumothorax, and chylothorax (3). An obstructive pattern, with air trapping, and a reduction in the diffusion capacity of the lungs for carbon monoxide (DL_CO_) are the most common abnormalities found during pulmonary function tests (PFTs) (4, 5). Functional impairment in subjects with LAM is frequently associated with exercise limitation (6, 7) that seems multifactorial, including ventilatory and gas exchange abnormalities, cardiovascular dysfunction, and muscle fatigue (7–9).

The 6-min walk test (6MWT) is a submaximal exercise test that is widely used to objectively assess the functional exercise capacity in patients with moderate-to-severe pulmonary disease (10), including LAM (7, 11). Although the distance walked is the primary outcome obtained during the 6MWT, other indexes that incorporate desaturation during the test, such as the desaturation–distance ratio (DDR), have been developed. The DDR is the ratio of the desaturation area to the distance walked. DDR has been considered predictive of morbidity and mortality in patients with other respiratory conditions, such as chronic pulmonary obstructive disease (COPD), pulmonary arterial hypertension, idiopathic pulmonary fibrosis, LAM, and those on the waiting list for lung transplantation (12–14). In addition, DDR is associated with pulmonary function and peripheral oxygen saturation (SpO_2_) in patients with interstitial lung diseases (ILDs) (15).

The incremental shuttle walk test (ISWT) is a field test that has been commonly used to quantify maximal exercise capacity. It is already known that 6-min walking distance (6MWD) has a good linear relationship with maximal exercise capacity (VO_2_peak); however, most patients reach a ceiling effect during 6MWT (16). Likewise, previous studies demonstrated that 6MWT might not properly evaluate physical capacity in patients with LAM (7, 11, 17). Therefore, other field tests should be tested in this population. The ISWT is a more standardized test that has been used to quantify exercise capacity in patients with chronic respiratory diseases leading to similar physiological responses than the cardiopulmonary exercise test (CPET) (10). However, the performance of patients with LAM and DDR values during the ISWT remains unknown. Therefore, our objectives were to assess DDR during ISWT and 6MWT and evaluate whether DDR is associated with lung function parameters.

## Methods

### Trial Design and Participants

This cross-sectional single-center study was conducted from September 2018 to January 2020 in a cohort of women with LAM from the ILD outpatient clinic of the Pulmonary Division from a tertiary university hospital in São Paulo, Brazil. The diagnosis of LAM was based on the current guidelines (3, 18) that include pulmonary function, computed tomography, and serum analysis. The protocol was approved by the Hospital Research Ethics Committee (90196617.1.0000.0068), and all patients signed an informed consent form. The patients were clinically stable (no exacerbation/hospitalization for the last 6 weeks) (18), and they maintained peripheral resting oxygen saturation (SpO_2_) of ≥ 88% in room air. The exclusion criteria were: supplementary oxygen use, other chronic respiratory diseases, musculoskeletal disorders or uncontrolled heart disease, pregnancy, lung transplantation, or any other disabling condition that could interfere with the tests.

### Experimental Design

The patients were evaluated during two nonconsecutive visits (maximum 7 days apart). During visit 1, the clinical and anthropometric data were obtained, and the subjects performed PFTs. After recovery, subjects were randomly assigned (http://www.randomization.com) for 6MWT or ISWT by an investigator not involved in the assessments. The allocations were sealed in opaque envelopes. If the subject performed the 6MWT during visit 1, the ISWT was performed during visit 2, and vice versa. DDR was evaluated during both tests.

### Measurements

#### Clinical and Anthropometric Evaluations

The following data were obtained: age, weight, height, identification and contact information, pathological antecedents, presence of comorbidities, and medication use.

#### Pulmonary Function Tests

Spirometry and body plethysmography (Bodystik Geratherm Respiratory GmbH, Bad Kissingen, Germany) were performed to obtain lung volumes (forced expiratory volume in 1 s, FEV_1_, and residual volume, RV), capacities (inspiratory capacity, IC; forced vital capacity, FVC; functional residual capacity, FRC and total lung capacity, TLC), and DL_CO_. The predicted values were based on the Brazilian population (19–21). The obstructive pattern, air trapping, and reduced DL_CO_ were defined according to the American Thoracic Society/European Respiratory Society criteria (22).

#### Peripheral Muscle Strength

Quadriceps strength was measured with a load cell integrated into a circuit in a chair fixed on a wooden plank. The load cell was previously calibrated and attached to the base of the chair with an inextensible strap. One side of the strap was fixed in the right ankle and the other in the load cell, keeping the knee flexed at 90°. During the test, the patient was asked to cross the arms on the chest and extend the knee. Three consecutive 5-s efforts were made at 30-s intervals, with visual feedback and verbal encouragement from the investigator. The maximum value was used in the analysis (23).

#### Dyspnea and Leg Fatigue Perception

The modified Borg scale was used to evaluate the intensity of dyspnea and leg fatigue, by quantifying the effort during the exercise—within a range from 0 to 10 points, where 0 indicated the absence of symptoms and 10 indicated the worst perception of dyspnea and leg fatigue (24).

#### Field Exercise Tests

##### Six-Minute Walking Test

The patient was asked to walk as far as possible along a 30-m corridor for 6-min. The 6-min walk distance (6MWD) was obtained at the end of the test (25). The predicted values for the distance walked were based on the Brazilian population (26). The tests were discontinued if SpO_2_ decreased below 80% (10). Before and after the test, heart rate (HR), blood pressure (BP), minimum SpO_2_ maintained for at least 10 s, dyspnea, and fatigue symptoms (Borg scale) were assessed. It was considered desaturation if SpO_2_ decreased by 4% from the basal level (27).

##### Incremental Shuttle Walking Test

The ISWT was conducted in an unobstructed and quiet 10-m corridor. The walking speed was determined using a standardized audio signal (beep) that started at 0.5 m/s and was progressively increased by 0.17 m/s every minute for a maximum of 20-min. The ISWT was terminated when the patient indicated that she could not continue or if the operator observed that the patient could not sustain the speed and cover the distance to the cone before the beep (28). SpO_2_ <80% was considered as the criterion for test discontinuation (10). Before and after the test, the HR, BP, minimum SpO_2_ maintained for at least 10 s, dyspnea, and fatigue symptoms (Borg scale) were assessed. Desaturation was characterized by a decrease in SpO_2_ of 4% from the basal level (27).

#### Desaturation–Distance Ratio

**Figure 1:**
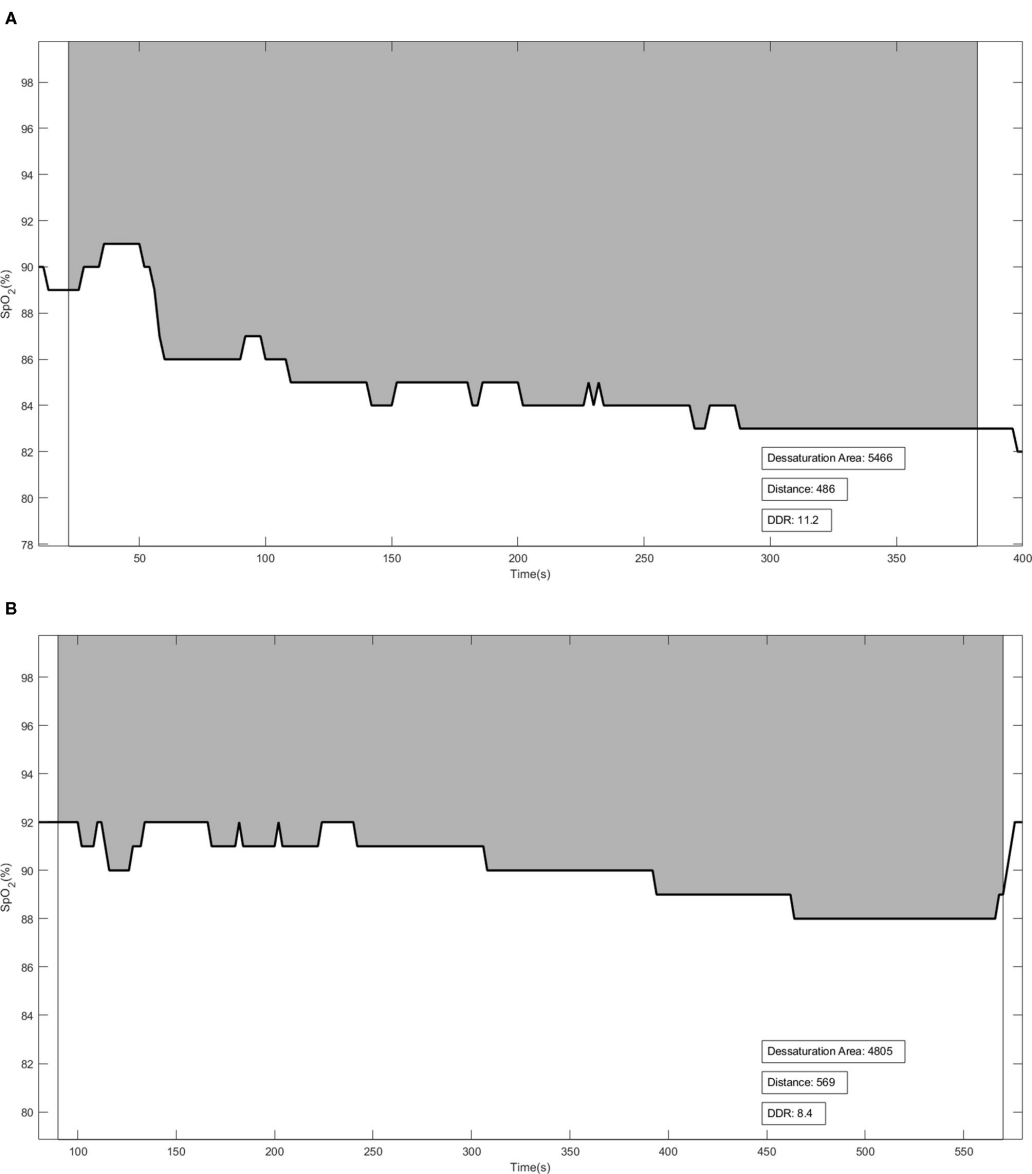
Illustrative figures of the Desaturation-Distance Ratio (DDR) during the 6-min walking test (6MWT) **(A)** and incremental shuttle walking test (ISWT) **(B)**. The solid line represents the oxygen desaturation during the test. DDR was calculated using the ratio between DAO_2_ [the gray area—obtained by subtraction between each recorded SpO_2_ at every 2 s from 100% (maximal SpO_2_)] and the distance walked. Distance in meters.

The DDR was the ratio of the desaturation area to the distance walked during the exercise tests. DDR was previously described by Pimenta et al. (15), who considered desaturation and distance walked as equally important variables for pulmonary functional assessment. During the 6MWT and ISWT, the patient used at pulse Holter oximeter (Nonin WristOxH 3100, Plymouth, MN, USA) to record SpO_2_ and HR every 2 s. All SpO_2_ values were obtained and recorded using software (nVISIONH, Plymouth, MN, USA). The desaturation area graph was plotted (DAO_2_, desaturation vs. time). DDR was calculated as the ratio of oxygen desaturation area (area under the curve) to the distance walked by the patient (DAO_2_/distance walked; Figures 1A,B). The patients who performed worst during the tests had higher DDR values.

### Statistical Analysis

Data are reported as the mean ± SD for variables with a parametric distribution or as the median (25–75% interquartile range) for the variables with a non-parametric distribution. The Pearson correlation coefficient was used to evaluate the association between the DDRs during the 6MWT (6MWT-DDR) and ISWT (ISWT-DDR) (dependent variables) and selected functional parameters (FEV_1_/FVC, FEV_1_/FVC%pred, FVCliters, FVC%pred, FEV_1_liters, FEV_1_%pred, FEV_1_/FVC%, TLCliters, TLC%pred, Ratio RV/TLC and RV/TLC%pred, DL_CO_ absolute, DL_CO_%pred). The linear correlation (*r*) was considered weak (from −0.3 to 0.3), moderate (from −0.5 to −0.31 or 0.31 to 0.5), strong (from −0.9 to −0.51 or 0.51 to 0.9), or very strong (from −1.0 to −0.91 or 0.91 to 1.0), according to Cohen's classification (29). A forward multiple linear regression analysis was performed, involving variables with linear correlation (*p* < 0.2). The best predictive models were constructed using the best independent coefficient. Receiver operating characteristic (ROC) curves were used to evaluate the sensitivity and specificity of the DDR for every field test. The optimal cut-point was calculated to predict airway obstruction (%FEV_1_ <80%) (19), reduced DL_CO_ (DL_CO_ <75%) (22), and air trapping (RV/TLC > 120%) (22). The optimum cutoff points were defined according to the Youden index (30). The level of significance was set at 5% (*p* < 0.05). The data were analyzed using Sigma Stat version 3.5 (Systat Software, Inc., San Jose, CA).

## Results

Sixty women were invited. Twenty declined to participate because they lived far from the hospital and would not honor the second appointment. Therefore, 40 women were included, and their clinical, anthropometric, and functional data are presented in Table 1. On average, patients showed peripheral muscle strength weakness (58.7% of the predicted). Obstructive pattern, air trapping, and reduced DL_CO_ were found in 60, 57, and 15% of patients, respectively. Twenty-seven patients (67%) were considered as “desaturators” (decrease of >4% from basal SpO_2_) during the 6MWT, and 36 patients (90%) presented with desaturation during the ISWT. Nineteen (47.5%) patients were not able to reach the speed imposed by the audio signal during the ISWT, and 21 (52.5%) patients were limited by their symptoms (16 due to dyspnea and 5 due to fatigue). The DDRs obtained during the 6MWT and ISWT were 6.6 (3.8–10.9) and 8.3 (6.2–12.7), respectively.

**Table 1:** Baseline anthropometrical, clinical, and functional characteristics of the patients with LAM.

**Variables**	***N*** **= 40**	
**Anthropometric data**		
Age (years)	46.60 (1.07)	
Weight (kg)	67.40 (14.4)	
Height (m)	1.60 (0.06)	
BMI (kg/m^2^)	26.60 (5.30)	
**Peripheral muscle strength**		
Quadriceps strength (kgf)	24.58 (8.21)	
Quadriceps strength (%)	58.7 (18.80)	
**Pulmonary function tests**		
FEV_1_ (l)	2.14 (0.54)	
FEV_1_ (% pred) and FVC (% pred)	75.45 (19.33)	
FVC (l)	2.95 (0.58)	
FEV1 (% pred) and FVC (% pred)	88.2 (19.27)	
FEV_1_/FVC (%)	72.63 (12.34)	
DL_CO_ (ml/min/mmHg)	17.31 (4.93)	
DL_CO_ (%pred)	72.12 (20.65)	
RV (l)	1.85 (1.00)	
RV (%pred)	112.5 (44.57)	
RV/TLC (%)	36.61 (10.83)	
RV/TLC (%pred)	121.82 (37.16)	
**Field exercise tests**	**6MWT**	**ISWT**
Distance (m)	516.70 (63.90)	452.70 (139.30)
Distance (%pred)	95.10 (17.80)	84.70 (22.0)
SpO_2_ basel (%)	95.60 (1.90)	95.90 (1.90)
SpO_2_ minimal (%)	89.40 (1.90)	86.20 (5.0)
DDR	6.6 (3.8–10.9)	8.3 (6.2–12.7)
Borg D (score)	2 (0.2–4)	4 (3–7)
Borg F (score)	2 (0.7–3)	3 (2–5)
HR (bpm)	107.10 (21.0)	142.20 (23.0)
Desaturation during test (%/total)	67/40	90/40

*Data presented as mean (standard deviation), except DDR and Borg, presented in median (25–75%, interquartile range). BMI, body mass index; FEV_1_, Forced expiratory volume in the first second; FVC, forced vital capacity; DL_CO_, carbon monoxide lung diffusion; RV, residual volume; TLC, total lung capacity; l, liters; min, minutes; kg, kilograms; m, meters; ml, milliliters; mmHg, millimeters of mercury; pred, predicted; 6MWT, 6-min walk test; ISWT, incremental shuttle walk test; SpO_2_, peripheral oxygen saturation; DDR, desaturation–distance ratio; Borg D, Borg dyspnea; Borg F, Borg fatigue; HR, heart rate; bpm, beats per minute*.

**Figure 2:**
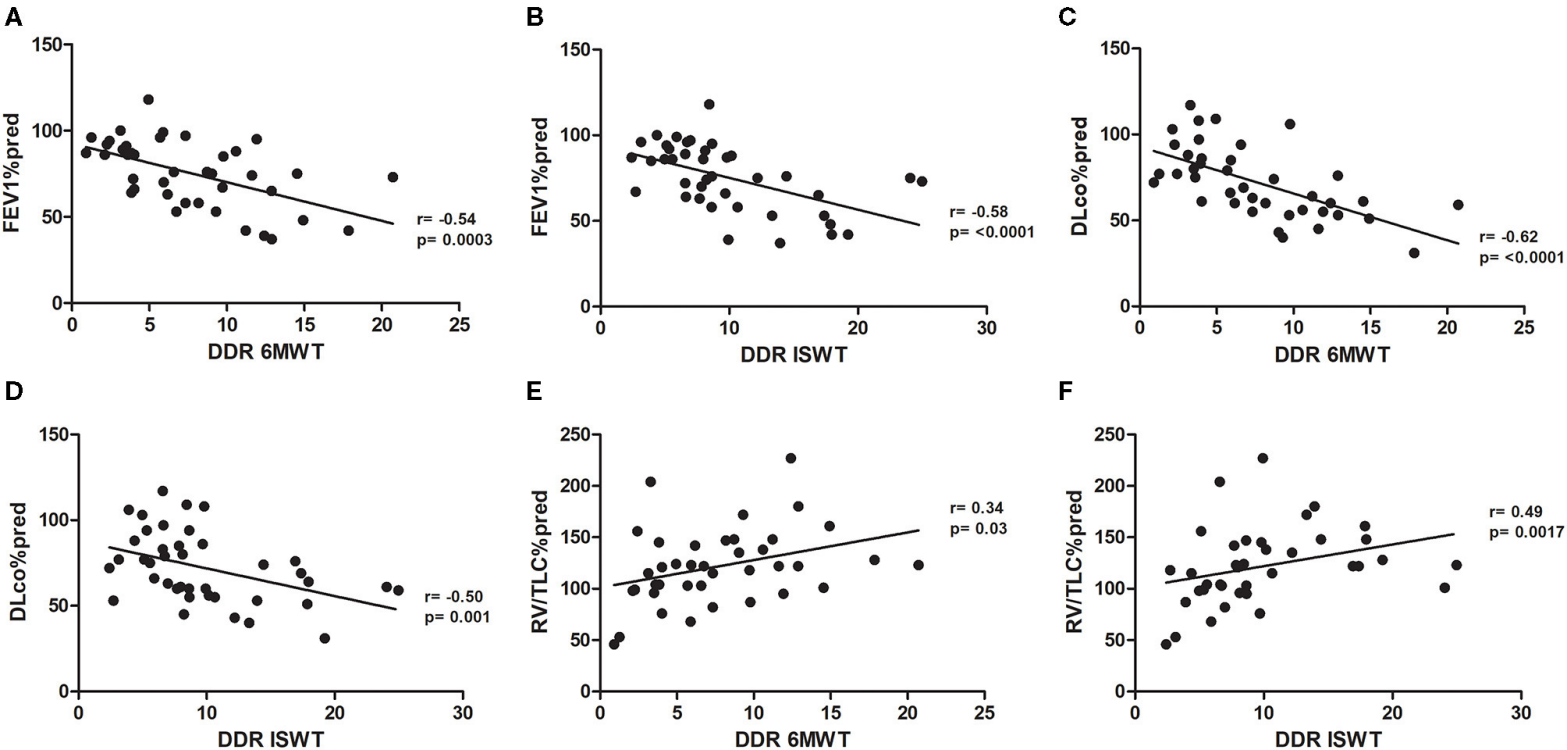
**(A)** Pearson's correlation between FEV1%pred and DDR 6MWT. **(B)** Pearson's correlation between FEV1%pred and DDR ISWT. **(C)** Pearson's correlation between DL_CO_%pred and DDR 6MWT. **(D)** Pearson's correlation between DL_CO_%pred and DDR ISWT. **(E)** Pearson's correlation between RV/LTC%pred and DDR 6MWT. **(F)** Pearson's correlation between RV/LTC%pred and DDR ISWT. FEV1%pred, forced expiratory volume in 1 s as a percentage of predicted; DDR, desaturation distance ratio; 6MWT, 6-min walking test; ISWT, incremental shuttle walking test; DL_CO_, diffusing capacity of the lung for carbon monoxide as a percentage of predicted; RV, residual volume; TLC, total lung capacity.

There were significant linear correlation between the DDR during the 6MWT and ISWT and the independent variables FEV_1_ (*r* = −0.54, *p* < 0.001, and *r* = −0.58, *p* < 0.001, respectively), DL_CO_ (*r* = −0.62, *p* < 0.001, and *r* = −0.50, *p* < 0.001, respectively), and RV/TLC (*r* = 0.34, *p* = 0.03, and *r* = 0.49, *p* < 0.001, respectively; Figures 2A–F).

The results of the ROC analysis show a high accuracy (area under the ROC curve [AUC] > 0.7) of 6MWT-DDR and ISWT-DDR for predicting airway obstruction (%FEV_1_ <80%), reduced lung diffusion (D_LCO_ <75%), and air trapping (RV/TLC > 120%; Table 2).

**Table 2:** The optimum cutoff points and ROC curve parameters for prediction of lung function and DDR in patients with LAM.

	**Cutoff point**	**Sensitivity**	**Specificity**	**AUC**	**95%CI**
**FEV_1_< 80%**					
6MWT-DDR	5.9	0.86	0.78	0.85	0.73–0.97
ISWT-DDR	8.5	0.73	0.83	0.85	0.74–0.97
**DL_CO_< 75%**					
6MWT-DDR	6.7	0.82	0.89	0.87	0.74–0.99
ISWT-DDR	9.9	0.59	0.94	0.78	0.64–0.93
**RV/TLC > 120%**					
6MWT-DDR	7.8	0.62	0.78	0.73	0.57–0.89
ISWT-DDR	7.3	0.91	0.67	0.82	0.68–0.96

*ROC, Receiver Operating Characteristic; DDR, Desaturation Distance Ratio; LAM, Lymphangioleiomyomatosis; AUC, Area under the curve; CI, Confidence Interval; FEV_1_, Forced Expiratory Volume in the first second of the Expiration (airway obstruction); 6MWT, 6-min walking test; ISWT, Incremental Shuttle Walking Test; DL_CO_, Diffusion Capacity of the Lungs for Carbon Monoxid (reduced lung diffusion); RV/LTC, Residual Volume/Lung Total Capacity (air trapping)*.

The best multivariate association models were constructed using the variables with the best independent coefficients of determination (*R*^2^). DDRs during 6MWT (DDR_6MWT_) and ISWT (DDR_ISWT_) for both models included only FEV_1_ (%pred) and DL_CO_ (%pred) and air trapping as a dichotomic variable (where RV/TLC>120% = 1; RV/TLC <120% = 0) were independent variables. In a stepwise multiple linear regression model, the derived prediction equations were as follows:

$$\begin{array}{l} \text{DD}{\text{R}}_{6\text{MWT}}=18.66-\left(0.06\;\times \text{ FE}{\text{V}}_{1}\text{\%}\right)-\left(0.10\;\times \text{ D}{\text{L}}_{\text{CO}}\text{\%}\right)+\left(1.54\;\times \text{ air trapping}\right),\\ {\text{R}}_{\text{adjust}}^{2}=0.43 \end{array}$$

$$\begin{array}{l} \text{DD}{\text{R}}_{\text{ISWT}}=18.84-\left(0.09\;\times \text{ FE}{\text{V}}_{1}\text{\%}\right)-\left(0.05\;\times \text{ D}{\text{L}}_{\text{CO}}\text{\%}\right)+\left(3.10\;\times \text{ air trapping}\right),\\ {\text{ R}}_{\text{adjust}}^{2}=0.33 \end{array}$$

## Discussion

To the best of our knowledge, this is the first study to investigate the roles of DDR in a general population of patients with LAM during 6MWT and ISWT and correlate it with functional parameters. The performance of patients with LAM during ISWT was also evaluated for the first time. Our results demonstrated that DDR obtained during the submaximal (6MWT) and maximal (ISWT) exercise tests were associated with the severity of pulmonary impairment, air trapping, and reduced DL_CO_ in patients with LAM. Additionally, our patients had satisfactory exercise capacities during both tests.

We included 40 women, which can be considered a relevant sample considering that LAM is a rare disease that affects ~5 persons per million adult women (1). Our patients were classified as having good exercise capacities during the 6MWT and ISWT (~95 and 85% of predicted, respectively). The 6MWT performance in our patients was similar to that observed in previous studies, ranging from 89 to 97% of the predicted distance during the 6MWT in patients with LAM with similar disease severity (7, 11). However, no previous study has assessed the performance of patients during the ISWT.

The DDR is based on the two main variables obtained during the 6MWT, the distance walked, and the decrease in SpO_2_ evaluated at regular intervals during the test (15). DDR is a more informative indicator for assessing exercise performance than oxygen desaturation, or the distance walked in isolation. Other advantages of DDR include its assessment through simple and low-cost tests (6MWT and ISWT), and easy application. DDR has been previously evaluated in patients with ILDs and COPD, and it has demonstrated associations with pulmonary function parameters (13, 15, 31). Fujimoto et al. (13) showed that DDR was highly predictive of the degree of emphysema and the enlargement of the pulmonary artery on computed tomography scans in COPD patients.

Oxygen desaturation during 6MWT is associated with a worse prognosis and greater mortality, and it has a good correlation with functional variables at rest, such as FVC, DL_CO_, and TLC in patients with ILDs (32–34). However, no previous study has evaluated the role of DDR in predicting disease progression and survival in ILDs, including LAM. Pimenta et al. (15) found that DDR was correlated with functional parameters, including DL_CO_ (%pred), FVC (%pred), and FEV_1_ (%pred). The authors included 15 patients with LAM and DDR in this subgroup, which was similar to that found in our study (6 vs. 6.6, respectively) (15). A previous study demonstrated that DDR was correlated with the severity of pulmonary cysts on high-resolution computed tomography (*r* = 0.77) in patients with LAM, reinforcing its potential role in evaluating disease severity (12). However, no study has assessed the association between DDR and pulmonary function parameters exclusively in LAM.

Our results demonstrated that DDR is associated with airway obstruction (FEV_1_), air trapping (RV/TLC), and DL_CO_ during the 6MWT and ISWT in patients with LAM. Previous investigations have shown that reduced exercise performance in cardiopulmonary exercise testing (CPET) is associated with such functional abnormalities in LAM (7, 8). The main mechanisms determining exercise cessation include ventilatory limitation, gas exchange impairment, peripheral muscle fatigue, and pulmonary hypertension (7, 8). However, no study has compared DDR with data obtained during CPET.

Obstructive pattern and reduced DL_CO_ were commonly observed in our patients, and they were predictors of DDR evaluated during the 6MWT and the ISWT, besides air trapping. Two DDR prediction equations were obtained, DDR-6MWT ($R_{\text{adjust}}^{2}$ = 0.43) and DDR-ISWT ($R_{\text{adjust}}^{2}$ = 0.33), based on functional abnormalities that were not previously described. The lung function findings observed in our study were similar to those observed by Li et al. (17) in Chinese patients who presented a mean FVC and FEV_1_ of 91%pred and 72%pred, respectively.

In patients with moderate and severe COPD, the distance covered during the ISWT is significantly associated with pulmonary function parameters, such as vital capacity and airway obstruction (FEV_1_), as well as health-related quality of life (35). According to Yildiz et al. (36), the ISWT distance is significantly associated with FEV_1_ (*r* = 0.65) and FVC (*r* = 0.54) in adults with bronchiectasis. The 6MWT is considered a submaximal field test aimed to assess functional capacity by measuring distance walked within a controlled duration. On the other hand, the ISWT is considered a maximal field test in which patients perform exercises until exhaustion. We are not aware of studies on ISWT in patients with LAM. However, the 6MWT and ISWT presented similar results relative to the predicted values (~90%, Table 1). In addition, the association between the 6MWT and ISWT with lung tests were similar.

Our study has some limitations. Our study was performed in only one center; however, our center is a referral center for LAM in Brazil, and it follows patients from different regions with different severities. We included 40 patients that can be considered significant due to the rarity of the disease. Another limitation is the insufficiency of the sample size for stratifying DDR predictions by age, impairment, or physical activity level. Also, no statement can be made regarding patients on oxygen. Finally, it is important to compare the performances during the ISWT and cardiopulmonary exercise testing in patients with LAM.

## Conclusion

In summary, DDR is useful for functional evaluation during submaximal and maximal exercise tests (6MWT and ISWT) in patients with LAM, and it is associated with functional impairment, reduced DL_CO_, and air trapping. Future studies are necessary to establish the effectiveness of DDR for evaluating exercises, in comparison with CPET, and predicting disease progression, survival, and response to therapeutic interventions in LAM patients.

## Data Availability Statement

The raw data supporting the conclusions of this article will be made available by the authors, without undue reservation.

## Ethics Statement

The studies involving human participants were reviewed and approved by Comissão em Ética e Pesquisa do Hospital das Clínicas da Faculdade de Medicina da Universidade de São Paulo (CAPPesq). Protocol 90196617.1.0000.0068. The patients/participants provided their written informed consent to participate in this study.

## Author Contributions

DQ promoted the development of the study design, the scheduling of patient appointments, collecting, analyzing, and interpreting the data, as well as writing the article. CS assisted the research helping develop the study design, scheduling patient appointments, collecting, analyzing, and interpreting the data, as well as improving and developing the article. AA and MO promoted the development of the study design and the scheduling of patient appointments. HM assisted us during the process and interpretation of the DDR data. CRRC greatly contributed to develop the study design, analyse, and interpret the data, as well as to help the elaboration of the article. BB supported us by developing the study design, scheduling patient appointments, analyzing, and interpreting the data, as well as helping the later elaboration of the article. CRFC conducted our research and also provided insight and expertise in all stages of the study, from the concept to the design, data collection and analysis, data interpretation, improvements, and the development of the article. All authors contributed to the article and approved the submitted version.

## Conflict of Interest

The authors declare that the research was conducted in the absence of any commercial or financial relationships that could be construed as a potential conflict of interest.

## Publisher's Note

All claims expressed in this article are solely those of the authors and do not necessarily represent those of their affiliated organizations, or those of the publisher, the editors and the reviewers. Any product that may be evaluated in this article, or claim that may be made by its manufacturer, is not guaranteed or endorsed by the publisher.
